# e-Vita: design of an innovative approach to COPD disease management in primary care through eHealth application

**DOI:** 10.1186/s12890-016-0283-4

**Published:** 2016-08-17

**Authors:** E. P. W. A. Talboom-Kamp, N. A. Verdijk, C. M. G. Blom, L. M. Harmans, I. J. S. H. Talboom, M. E. Numans, N. H. Chavannes

**Affiliations:** 1 Public Health and Primary Care Department, LUMC, P.O. Box 9600, 2300 RC Leiden, The Netherlands; 2 SALTRO Diagnostic Centre, Mississippidreef 83, 3565 CE Utrecht, The Netherlands; 3 Zorgdraad Foundation, Wijnand van Arnhemweg 54, 6862XN Oosterbeek, The Netherlands

**Keywords:** eHealth, Self-management, COPD, Integrated disease management, Chronically ill, Telemonitoring, Primary care

## Abstract

**Background:**

COPD is a highly complex disease to manage as patients show great variation in symptoms and limitations in daily life. In the last decade self-management support of COPD has been introduced as an effective method to improve quality and efficiency of care, and to reduce healthcare costs. Despite the urge to change the organisation of health care and the potential of eHealth to support this, large-scale implementation in daily practice remains behind, especially in the Netherlands.

**Methods/Design:**

We designed a multilevel study, called e-Vita, to investigate different organisational implementation methods of a self-management web portal to support and empower patients with COPD in three different primary care settings. Using a parallel cohort design, the clinical effects of the web portal will be assessed using an interrupted times series (ITS) study design and measured according to changes in health status with the Clinical COPD Questionnaire (CCQ). The different implementations and net benefits of self-management through eHealth on clinical outcomes will be evaluated from human, organisational, and technical perspectives.

**Discussion:**

To our knowledge this is the first study to combine different study designs that enable simultaneous investigation of clinical effects, as well as effects of different organisational implementation methods whilst controlling for confounding effects of the organisational characteristics. We hypothesize that an implementation with higher levels of personal assistance, and integrated in an existing care program will result in increased use of and satisfaction with the platform, thereby increasing health status and diminishing exacerbation and hospitalisation.

**Trial registration number:**

NTR4098 (31-07-2013)

## Background

Chronic obstructive pulmonary disease (COPD) represents one of the main causes of morbidity and mortality, and worldwide nearly 3 million people die from COPD every year [1]. In the Netherlands, COPD was responsible for almost 5 % of the total deaths in 2011 [2]. More than 3 million people died worldwide of COPD in 2012, which is equal to 6 % of all deaths globally that year [3]. COPD is a highly complex disease to manage as patients show great variation in symptoms and limitations in daily life. This results in a position in the top ten of most expensive diseases for respiratory disease [2]. Within the last decade self-management support of COPD has been introduced as an effective method to improve quality and efficiency of care, and to reduce healthcare costs [4–6]. It has shown to improve the level of recognition of severe exacerbations [7]. Interventions to support self-management have shown reductions in hospital admissions and fewer sick days as a result of exacerbations [8, 9]. Studies have shown that eHealth interventions are effective in stimulating self-management. Patients are better able to cope with their illness at the time and place of their choosing, allowing them to adapt their lifestyle to their condition while eHealth support also reduces medical staff consultations [10]. The deployment of eHealth applications facilitates accessibility to healthcare, which in turn enhances the patients’ understanding of their disease, sense of control, and willingness to engage in self-management [11, 12]. Although patients’ attitudes and receptiveness towards eHealth applications are promising in certain groups of age and education [13–15], large-scale adoption of eHealth in daily practice is low. Despite the urge to change the organisation of health care and the potential of eHealth to support this, large-scale implementation in daily practice remains behind on predictions, especially in the Netherlands [16].

Low adoption of eHealth in daily practice may be explained by the varying successes of eHealth programmes [17–20]-sometimes with a negative impact on quality of care and clinical effects [21]. In addition, the field of eHealth assessment is relatively new. The evaluation of eHealth research has a number of difficulties regarding evaluation methods and challenges of technology itself (usability and privacy), environmental issues that pose special problems for eHealth researchers, and logistic or administrative concerns of the selected evaluation method [11]. Therefore specific frameworks have been developed for eHealth evaluation, including evaluation of eHealth over time and based on different development stages. For example, Kaufman et al. [22] suggest that evaluation of eHealth includes specification and needs of requirements, component development, integration of components, integration in clinical setting and routine use. There are also frameworks that suggest that eHealth should be evaluated from different point of views. For example, Yusof et al. [23] suggest that human, organisational, and technological aspects and net benefits are essential components of eHealth evaluation.

**Fig. 1:**
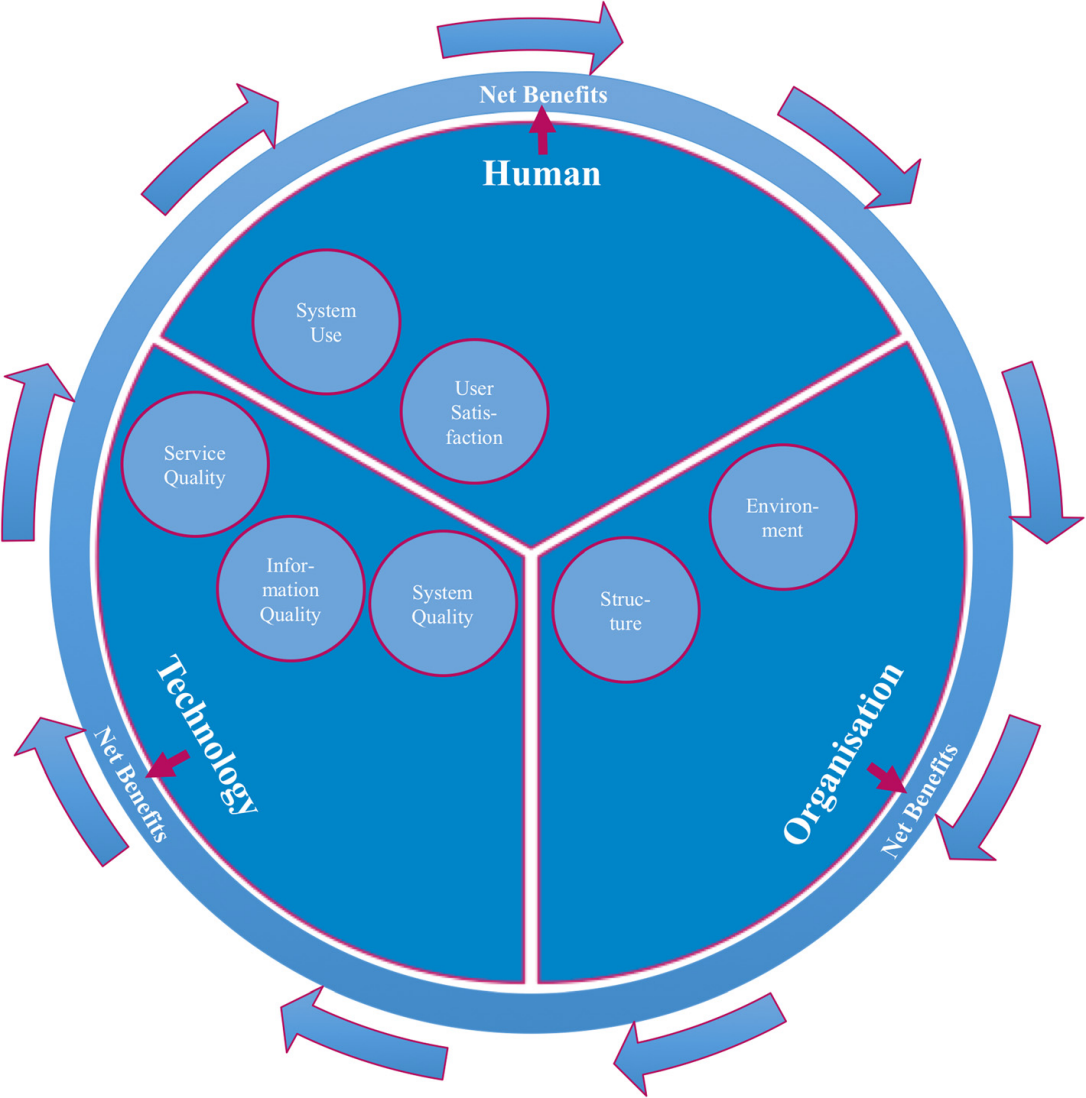
Model based on Yusof [22]: different aspects of eHealth evaluation

eHealth shows great potential for effective COPD management. Despite the difficulties of evaluation, research of eHealth interventions is highly valuable for further adoption of eHealth in daily practice. Positive as well as negative results are needed to improve quality, utility and effectiveness, to minimize the likelihood of harm, to promote innovation, conserve resources, encourage participation, to promote confidence among users, and to promote a positive public image [24]. Therefore we designed a multilevel study to investigate implementation of a self-management web portal to support patients with COPD in primary care. As the web portal provides continuous education and contact with health care professionals, we expect it to help patients to better recognize and self-manage exacerbations in an early phase, and thereby increase health status and diminish exacerbation and hospitalisation. In this ongoing study, called e-Vita, we compare different organisational implementation methods in different primary care settings. We aim to investigate 1) the effect of self-management through eHealth on clinical outcomes and 2) the relationship between technological and organisational factors on the one hand and system use and user satisfaction on the other hand. We will therefore evaluate implementation and net benefits from human, organisational and technical perspectives (Fig. 1).

We expect to publish the first results at the end of 2016.

## Methods/Design

Our primary aim is to investigate the effect of use of patient portals on clinical outcomes in primary care COPD patients (net benefits, Fig. 1). In addition, we aim to investigate the optimal organisational implementation method of the platform. Therefore we will compare different organisational implementation methods in different care settings, and evaluate their use and user satisfaction. We hypothesize that an implementation setup with greater personal support, integrated into an existing care programme will be preferred by patients and therefore be more likely to be used.

As discussed in the introduction, the evaluation of eHealth is a challenge because of the different views to evaluate from (clinical, technical, and organisational). Moreover, eHealth is not a classical clinical isolated intervention (in this case a platform) with one output (here CCQ). Instead it affects care processes, communication and patients behaviour, Finally we have a multilevel purpose to not only investigate effects but also organisational implementation methods. Therefore we made a design for a quality improvement intervention. In this study we aimed to include the importance of integration in the daily practice of primary care. Therefore we chose an implementation study [25]. We designed a method to promote the uptake of our research findings into routine primary healthcare; with this design we aim at studying the influences on healthcare professionals and patient behaviour and at evaluating the process by which effects are achieved.

Because the most powerful studies are prospective studies, we chose a prospective parallel cohort design. We asked three primary care groups to invite their COPD patients for the e-Vita study. Because there are several differences between the groups (Fig. 3), we can not compare the COPD cohorts by combining data across three different groups. We chose for an interrupted time series (ITS) design to evaluate clinical outcomes (CCQ) within each group. In ITS studies, data are collected at multiple time points before and after an intervention in order to detect whether or not the intervention has a significantly greater effect than any underlying secular trend [26]. ITS can detect changes that are delayed or intermittent. It can also determine if the change is permanent or temporary. In addition, it allows evaluation of variables which are changing before the intervention, for instance, by comparing slopes of trend lines before and after the intervention. Finally, ITS makes it easier to control for confounding variables and regression to the mean [27]. The ITS will be performed according to guidelines of the EPOC Cochrane group [25]. Although well-conducted randomised trials provide the most reliable evidence on the effectiveness of interventions, they are not feasible for our setting of an implementation design with organisational changes in a real-life health care system within three different care groups with different demands. An advantage of an ITS design is that it allows for the statistical investigation of potential biases in the estimate of the effect of the intervention.

In addition to clinical outcomes, we also want to investigate the effect of different organisational implementation methods. Therefore we will implement the platform in group 1 and 2 using different methods. Differences will be measured using a parallel cohort design. To be certain that intervention groups will be balanced in known and unknown prognostic factors in the long run we will use randomisation within group 1 and 2. To rule out human influences we will randomise online for the level of support.

**Fig. 2:**
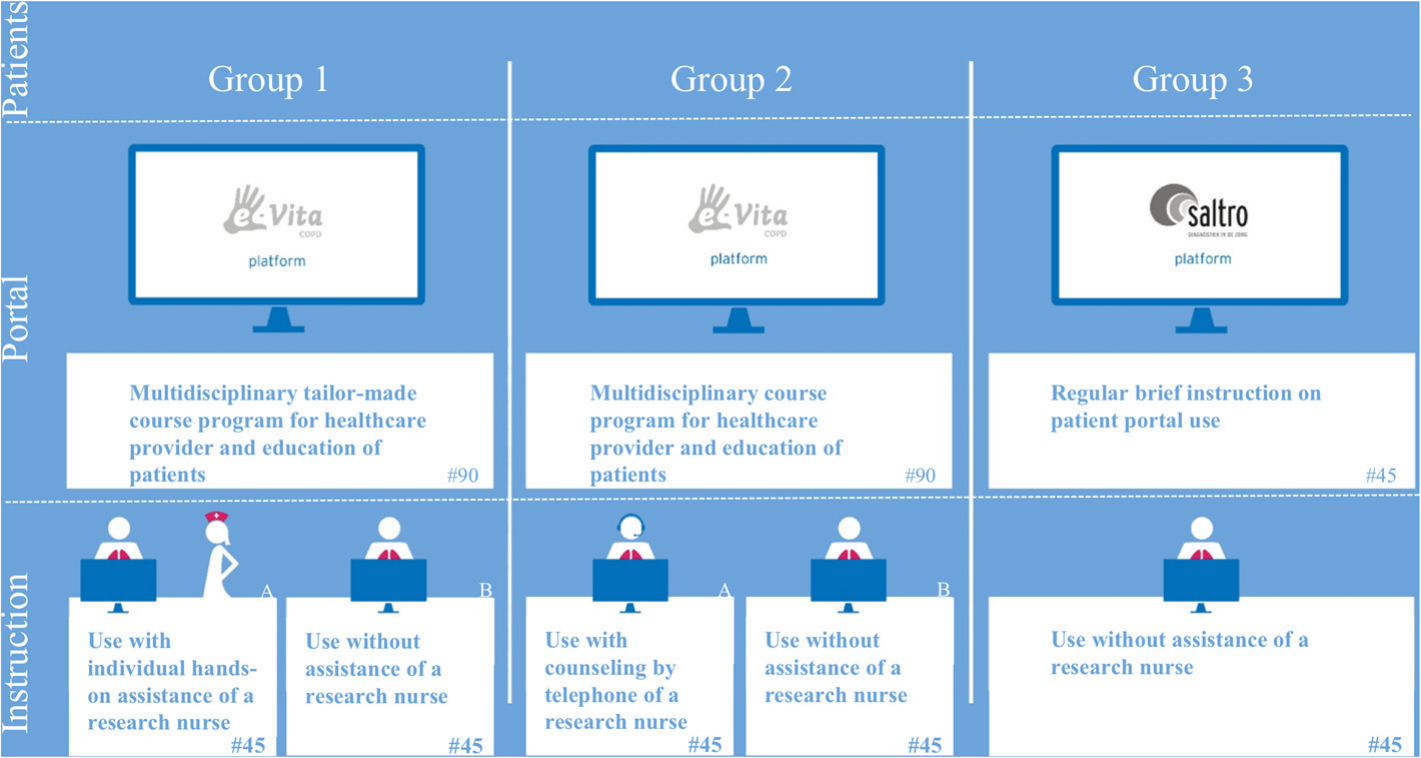
Research design for the different groups and interventions of e-Vita COPD

In this study we combine different study methods within one research to investigate organisational implementation methods and net benefits of eHealth interventions from human, organisational, and technical view. To do so, we created a unique study that enables us to simultaneously investigate clinical effects, as well as effects of different organisational implementation methods whilst controlling for confounding effects on an organizational level. In Fig. 2 the combined study design is shown.

Organisational and technical differences are depicted in Fig. 2: three different care groups and two different web portals are included. Two different methods of implementation are distinguished within group 1 and 2: one with high level of personal assistance and one with low level of personal assistance. All patients in group 1 and 2 start with a personal instruction by the primary care nurse during a regular control visit. Patients will be randomly subdivided in two groups by computer by research assistants (randomisation is computerised to prevent human influence) with high (a) and low (b) level of support. In group 1a, high level support implies home visits for patients by a research nurse who accompanies the use of the web portal. In group 2a, high level support implies telephone consultation for patients by a research nurse who accompanies use of the web portal. In group 1b and 2b low level support implies that the primary care nurse shows the patient only once how to use the web portal (the usual organisational implementation method in daily practice) without any follow-up instruction. In group 3 the web portal is offered as free use: patients will receive instructions from the web portal itself. There will be no active support from caregivers or research nurses.

**Fig. 3:**
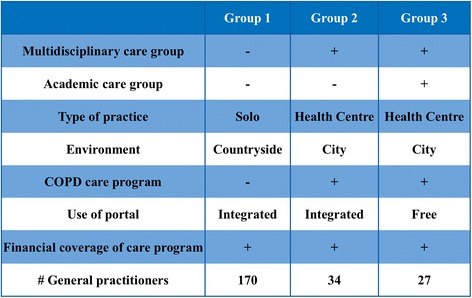
The different characteristics of group 1, 2, and 3

The groups differ in organisation, area, use of the web portal, and integration of the portal in a COPD disease management program (Fig. 3); these characteristics are based on Dutch reports of care groups [28]:

1. Group 1 will start with COPD disease management simultaneous to implementation of the web portal. The web portal is integrated in the disease management program (integrated use).
2. Group 2 is used to working with a COPD disease management program. They will start with the web portal, which is integrated in their own disease management program (integrated use).
3. Group 3 is used to working with a COPD disease management program. They will start with the web portal, but the web portal is not integrated in their own disease management program (free use).

**Fig. 4:**
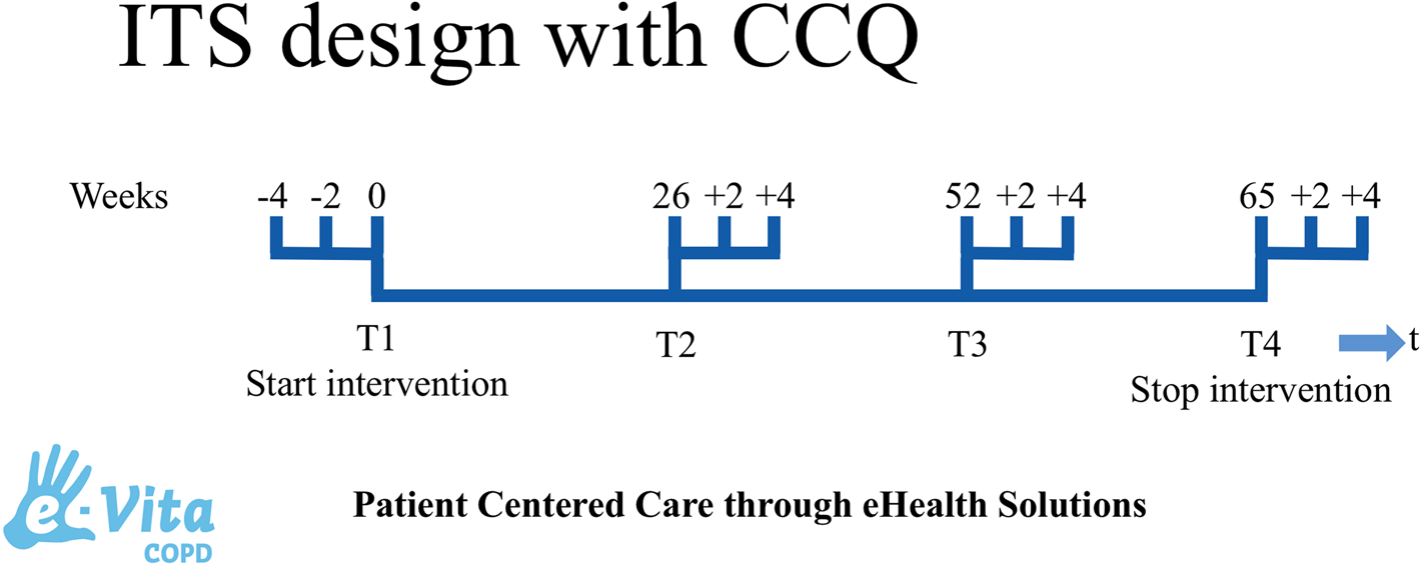
ITS design with CCQ

As was described above, the implementation of the portal will be studied using a prospective parallel cohort design. The clinical effects of the web portal will be investigated using an interrupted times series (ITS) study design (Fig. 4) and measured according to changes in health status with the Clinical COPD Questionnaire (CCQ, see Appendix 1). ITS design includes multiple observations over time that are ‘interrupted’ by interventions. The time intervals between the observations T1, T2, T3, and T4 are 6 months, to detect the change in CCQ in a trend and slope over the total period of time. The time intervals between the 3 measurements of each observation T1, T2, T3, and T4 is two weeks, based on the high responsiveness of CCQ [29]. The ITS will be performed according to guidelines of the EPOC Cochrane group [30]. The aim of an ITS design is to detect confounding trends by performing several measurements at specified time intervals, before and after the intervention. An advantage of an ITS design is that it allows for the statistical investigation of potential biases in the estimate of the effect of the intervention.

### Participants

Three health care groups participate in this study. Therefore, COPD patients of general practices in these care groups are eligible. More specifically, patients are eligible when they are diagnosed with COPD according to GOLD criteria (post-bronchodilator FEV1/FVC < 0.7) in accordance with the Dutch general practitioners (GPs) COPD Guidelines [31] and when they are treated for COPD in primary care. The study is intended to be inclusive rather than exclusive to achieve high external validity (applicability to daily practice). Patients are excluded if they are unable to fill in questionnaires, patients that have no access to internet, patients with terminal illness, immobile patients and patients with severe substance abuse.

### Recruitment of patients and non-participation analysis

We started by recruiting primary care groups; group 1, group 2, and group 3 decided to participate in this study because they wanted to contribute to a project with a possible healthcare improvement. The general practitioners that are part of the care groups could volunteer to participate in the study; a selection of them did.

Because general practices as well as patients are free to volunteer, bias might occur in our research group. We will determine the differences in clinical status between study participants (included patients) and non-participants (eligible patients) by CCQ questionnaire, as well as gender and age differences.

### Intervention

**Fig. 5:**
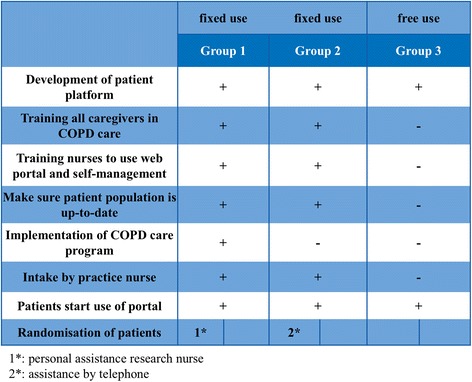
Interventions per group

In Fig. 5 all actions of the intervention are summarized:

1. In group 1 all caregivers (GPs as well as practice nurses) will be trained to provide COPD care according to an evidence based disease management program; subsequently they implement the COPD care program in their practices under supervision of a specialized nurse. Practice nurses will receive support by a research nurse to make sure their records of COPD patients are up-to-date and to prepare consultation with their patients according to the disease management program. In addition, all practice nurses will be trained to use the web portal and to communicate with patients according to the principles of self-management.
2. In group 2 all caregivers (GPs as well as practice nurses) will be trained shortly to ameliorate their skills in COPD care according to an evidence based disease management program that they already use. All practice nurses will be trained to use the web portal and to communicate with patients according to the principles of self-management. The training is developed and provided for by the e-Vita study group and is based on national and international guidelines.
3. In group 3 caregivers will not receive any training. Patients will receive a brochure how to use the web portal.

The type of intervention is adopted by the care groups after thorough consideration and discussion. The e-Vita study group determined the type of platform and the level of support.

Next, we will offer patients an online interactive care platform or web portal. The portal provides disease specific education and tips that fit their personal disease management program. In addition, the portal provides tools to report and monitor personal health goals, actions and health-related quality of life that can be shared with the patients’ own practice nurses. The portal in group 1 and 2 has better quality and more advanced possibilities for monitoring health goals with actions than the portal in group 3. The portal will be provided for a period of 15 months. Patients are informed by letter about the web portal. Patients in group 3 who agree to use the portal will receive instructions and log in information by e-mail. Patients in group 1 and 2 who agree to use the portal will be invited by their own practice nurses for intake. During intake the practice nurse defines a personal health goal together with the patient and gives instructions how and why to use the portal. Participants continue to receive regular COPD care by their GP and nurse practitioner according to the disease management program of the care group. Stable COPD patients visit their nurse practitioner yearly to check up on their disease management. The patient portal can be used by care professionals to prepare consultation or to monitor patients in-between their visits to their general practice.

Third, a subgroup of participants (1a and 2a) will receive extra support to use the portal by home visits or with instructions by telephone. During the home visit and consultation it will be checked if patients are able to log on the portal, if they understand the possibilities of the portal, and if they have started working on their personal health goal using the portal.

### Data collection

**Fig. 6:**
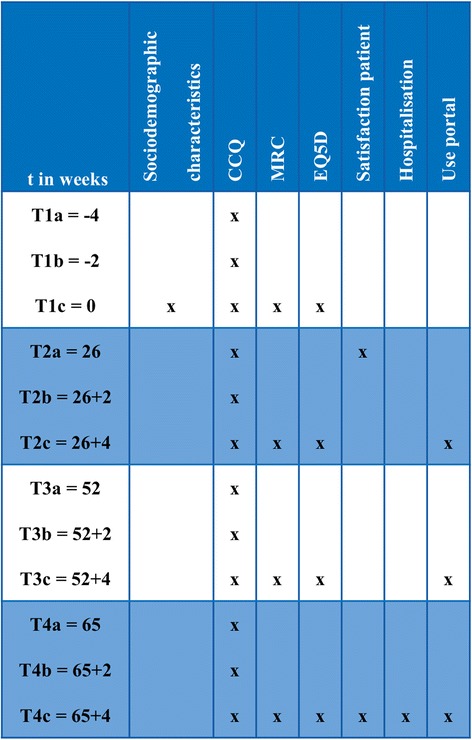
ITS measurements per interval

Data collection consists of self reporting questionnaires that are integrated in the portal. Therefore all data collection is provided digitally. In Fig. 6 the measurement schedule is visualized. There are four measurements in this study during a period of 16 months. Due to the ITS design, CCQs will be offered three times at each measurement.

### Outcome parameters

#### Primary outcome

The primary outcome is clinical and expressed as health status, measured using the CCQ. The CCQ was originally designed by Van der Molen and consist of 10 items with a 7-point Likert scale. The CCQ comprises 3 domains; symptom state (4 items), functional state (4 items) and mental state (2 items). Research showed that the CCQ is a reliable and valid questionnaire with Crohnbach’s alpha between 0.89 and 0.91 [29].

#### Secondary outcomes

From the human perspective we will measure the following outcomes:

##### Disability associated with breathlessness

This is measured using the Medical Research Council (MRC) breathlessness scale (see Appendix 2), which comprises five statements that describe almost the entire range of respiratory disability: from none (Grade 1) to almost complete incapacity (Grade 5). It can predict survival [32] and it is advocated as complementary to FEV_1_ in describing disability in those with COPD [33].

##### Quality of life (QoL)

QoL will be assessed using EuroQol-5D (EQ-5D). This questionnaire contains 5 items with a 3-point Likert scale. A higher score reflects higher quality of life. The EQ-5D comprises 5 levels: mobility, self care, daily activity, pain/discomfort and anxiety/depression. Research showed that the EQ-5D is a reliable and valid questionnaire [34]. The EQ-5D can be used to compute QALY’s, which are necessary to evaluate cost-effectiveness.

##### Adoption of the portal

Usage of the portal is monitored continuously by log files. User satisfaction is measured by purpose-designed questionnaires.

From the organisational perspective we will measure the following outcome:

##### Costs

In this study we include direct costs of the intervention and COPD care. Intervention costs include development costs of the patient platform and costs of the implementation process. Development costs are provided retrospectively by the owner of the portal. Implementation costs are administered by the research group and mainly include costs of home visits and interviews by telephone. The cost for COPD care (time of professional care) is extracted from the portal in which consultation in general practice will be registered in an agenda. In addition, hospitalisation (caused by COPD exacerbation) will be based on reports of the patient and practice nurse in the portal. All costs are based on regular tariffs in Dutch healthcare.

#### Determinants

##### Self-efficacy

Self-efficacy is measured using the Generalized Self-Efficacy Scale (GSES), which will be displayed at baseline. This 10-item questionnaire was designed in 1981 by Schwarzer [35]. The items are scored on a four-point scale, in which a higher score reflects higher self-efficacy. Research in 28 countries showed that Cronbach’s alpha varies between .76 and .90, of which mostly above .80. Self-efficacy is derived from the Social Cognitive Theory, which states that behavioral change is made possible by personal sense of control. Self-efficacy is the “belief in one’s capabilities to organise and execute the courses of action required to produce given attainments”. Research shows that self-efficacy is an important factor for self-management in behavioral change of the chronic ill, such as diabetics and cardiovascular patients. For example, in people with type 2 diabetes self-efficacy is an important factor influencing self-management behaviours; self-efficacy impacts adherence to treatment [36].

##### Sociodemographic characteristics

These are assessed by a purpose-designed online questionnaire. We include the following characteristics: age, socioeconomic status, marital status, and general use of online and digital products and services. Since decreased access to internet and decreased general health outcomes have been associated with lower socioeconomic status, minority racial/ethnic groups, older age, and poorer health we will include these characteristics in our study.

### Power calculation

Health status of patients with COPD generally decreases over time. Recent research on disease management programs in COPD in primary care shows that a general increase of 1.5 to 2.0 points (SD 0.75) in CCQ can be expected during a one year period [37]. In our study we offer patients a web portal in addition to their regular disease management program. We therefore expect that the regular deterioration in CCQ (from 1.5 to 2.0 points) will change to stabilisation of health status at 1.5 CCQ points. Hence, we expect a significant difference in health status of 0.5 points in patients using the portal (2.0-1.5 = 0.5 points). To measure significant differences in health status (>0.5 CCQ points) at 80 % power, SD 0.75 and *α* = 0.05, 37 patients must be included. Based on an estimated 20 % drop-out during the study, 45 (37/0.80) patients are needed. As we use two different organisational implementation methods within two of the care groups 2*45 = 90 patients must be included in those settings. In the third care group only one organisational implementation method (free use) is used. Hence, a total of 225 (90 + 90 + 45) patients are necessary to achieve sufficient statistical power.

### Data analysis

Along with our research design, analyses will be multilevel:

1. To investigate the effect of the use of the web portal on the primary clinical outcomes, the ITS data will be analysed. The preferred method to analyse ITS studies is a statistical comparison of time trends before and after the intervention. Time series analysis using ARIMA models is one way of analysing the data, but there are a number of statistical techniques that can be used depending on the characteristics of the data, the number of data points available and whether autocorrelation is present. The final choice for the method to analyse the data will be made when the total set of data is available after consultation of a statistician.
2. To investigate the effects of organisational aspects uptake of the portal will be analysed for care group 1, 2 and 3 using Chi Square tests (Fishers’ Exact Test for categorical variables and *F*-tests for continuous variables) and (repeated measures) ANOVA analyses. To analyse the effects of organizational aspects as described in the paper, the groups will be compared.
3. To investigate the effect of integration of the web portal in daily practice, outcomes within integrated (group 1 and 2) and the free use groups (group 3) will be analysed using Chi Square tests (Fishers’ Exact Test for categorical variables and *F*-tests for continuous variables) and (repeated measures) ANOVA analyses.
4. To investigate the effect of different organisational implementation methods, outcomes within groups with high level support (1a and 2a) and low level support (1b, 2b and 3) will be analysed using Chi Square tests (Fishers’ Exact Test for categorical variables and *F*-tests for continuous variables) and (repeated measures) ANOVA analyses.
5. To explore correlation between self efficacy, sociodemographic characteristics on the one hand and adoption of the portal and clinical outcomes on the other hand, Pearson product-moment coefficients will be calculated across all research groups.
6. To make a cost-effectiveness analysis, the direct costs of the different organisational implementation methods will be defined and analysed parallel to the effects of the portal in terms of Quality of Life (EQ-5D).

### Ethical principles

Participation in the study provides several benefits: increasing costs and deficit of health care professionals stress the need for efficient health care processes. Benefits of eHealth regarding clinical effects and costs have repeatedly been demonstrated, but extensive integration in clinical practice stays behind. This projects aims to explore organisational implementation methods for optimal integration of patient portals in primary care. Optimal integration stimulates patients in self-management and improves efficiency and accuracy administration and communication. We expect patients to improve their health status while decreasing health care use. Health care providers participating in these projects improve communication between different workers, adherence to guidelines, and thereby increase quality of care. The online portals will not be offered to patients who are unable to use the online portals. However, they will not be in disadvantage by receiving usual care.

## Discussion

COPD is one of the main causes of morbidity and mortality in the world. Worldwide nearly 3 million people die from COPD every year [1]. COPD is a highly complex disease to manage as patients show great variation in symptoms and limitations in daily life. An important treatment of COPD is empowerment of patients: self-management may reduce hospital admission and significantly improves health status [38]; it can diminish the impact of exacerbations on health status and tends to accelerate recovery [39, 40]. eHealth tools for COPD patients have potential to raise self-management to higher levels. Patients’ attitudes and receptiveness towards eHealth applications are promising [20] but lack of robust trials and inconclusive research results [17–20] make it impossible to draw firm conclusions about clinical effectiveness or cost effectiveness.

In this study we aim to empower COPD patients in primary care by providing a self-management web portal. We expect this portal to help patients to better recognize and self-manage exacerbations in an early phase, thereby increasing health status and diminishing exacerbation and hospitalisation. In addition we aim to provide practical insights into a successful implementation of patients portals in real-life primary care settings. We will therefore compare different organisational implementation methods. We expect that an implementation setup with greater personal support will result in increased use of the online program.

This e-Vita study has several strengths. To our knowledge this is the first study to combine different study designs that enable simultaneous investigation of clinical effects, as well as effects of different organisational implementation methods whilst controlling for confounding effects of the organisational characteristics. Our hypothesis is that in well-organised primary care groups with highly skilled and motivated nurses and doctors there will be a higher use of the portal and therefore better health status. Secondly, our web portal is integrated in real life care settings and will therefore provide practical insights and knowledge of eHealth in daily practice. Third, this study adds Dutch evidence to the existing body of knowledge which is important because local political and financial factors have a major impact on successful integration in daily practice [41]. This study also includes several limitations: from a technical perspective the development of the web portal is a difficult task due to lack of broad experience in the field. The technique of the web portal and decisions made during the design phase will largely affect our outcomes but are beyond the scope and influence of our study. From a human perspective, effects through self-management imply behavioural changes. Behavioural changes require time, whereas the study period is limited to 15 months. Furthermore, patients in a primary care setting have a low burden of disease. From an organisational perspective other projects in the primary care cooperation’s can influence the speed and thoroughness of the implementation of our web portal.

## Abbreviations

ANOVA, analysis of variance; CCQ, clinical COPD questionnaire; COPD, chronic obstructive pulmonary disease; EQ-5D, EuroQoL five dimensions questionnaire; FEV, forced expiratory volume; FVC, forced vital capacity; GOLD, Global Initiative for Chronic Obstructive Lung Disease; GP, general practitioner; GSES, generalized self-efficacy scale; ITS, interrupted times series; MRC, Medical Research Council; QALY, quality-adjusted life year; QoL, quality of life; SD, standard deviation; WMO, Wet Medisch-wetenschappelijk Onderzoek met mensen (Medical Research Involving Human Subjects Act)

## Appendix 1

**Table 1:** Please circle the number of the response that best describes how you have been feeling during the past week. (Only one response for each question)

During the past week, how often did you feel…							
	Never	Hardly	A few	Several times	Many times	Most of the time	All the time
1. Short of breath at rest?	0	1	2	3	4	5	6
2. Short of breath doing physical activities?	0	1	2	3	4	5	6
3. Concerned about your breathing getting worse?	0	1	2	3	4	5	6
4. Depressed (down) because of your breathing problems?	0	1	2	3	4	5	6
During the past week, how much of the time did you…							
5. Cough	0	1	2	3	4	5	6
6. Produce phlegm?	0	1	2	3	4	5	6
During the past week, how limited were you in these activities because of your breathing problems when doing…							
	Not limited at all	Very slightly limited	Slightly limited	Moderately limited	Very limited	Extremely limited	Totally limited or unable to do
7. Strenuous physical activities (such as climbing stairs, hurrying, doing sports)?							
8. Moderate physical activities (such as walking, housework, carrying things)?							
9. Daily activities at home (such as dressing, washing yourself)?							
10. Social activities (such as talking, being with children, visiting friends/relatives)?							

## Appendix 2

**Table 2:** ᅟ

Do you ever experience breathlessness?		
Which of the following statement applies most to you?		
O	I do not experience breathlessness	0
O	I am only troubled by breathlessness during strenuous exercise	1
O	I am only short of breath when hurrying on the level or walking up a slight hill	2
O	My breathlessness makes me walk slower on the level than most people my age, or results in the need to stop for breath after walking at my own pace after 15 minutes	3
O	I need to stop for breath for a few minutes after walking 100 meters on the level	4
O	I am too breathless to leave the house, or need to catch my breath when undressing	5
